# *Bacillus subtilis* promoter sequences data set for promoter prediction in Gram-positive bacteria

**DOI:** 10.1016/j.dib.2018.05.025

**Published:** 2018-05-13

**Authors:** Rafael Vieira Coelho, Scheila de Avila e Silva, Sergio Echeverrigaray, Ana Paula Longaray Delamare

**Affiliations:** a Rio Grande do Sul Federal Institute of Education, Science and Technology (IFRS), Farroupilha Campus, Farroupilha, RS, Brazil; b Biotechnology Institute, University of Caxias do Sul (UCS), Caxias do Sul, RS, Brazil

**Keywords:** Promoter sequences, *Bacillus subtilis*, SVM

## Abstract

This paper presents a prediction of *Bacillus subtilis* promoters using a Support Vector Machine system. In the literature, there is a lack of information on Gram-positive bacterial promoter sequences compared to Gram-negative bacteria. Promoter sequence identification is essential for studying gene expression. Initially, we collected the *B. subtilis* genome sequence from the NCBI database, and promoters were identified by their sigma factors in the DBTBS database. We then grouped the promoters according to 15 factors in 2 domains, corresponding to sigma 54 and sigma 70 of Gram-negative bacteria. Based on these data we developed a script in Python to search for promoters in the *B. subtilis* genome. After processing the data, we obtained 767 promoter sequences for *B. subtilis*, most of which were recognized by sigma SigA. To validate the data we found, we developed a software package called BacSVM+, which receives promoters as input and returns the best combination of parameters in a LibSVM library to predict promoter regions in the bacteria used in the simulation. All data gathered as well as the BacSVM+ software is available for download at http://bacpp.bioinfoucs.com/rafael/Sigmas.zip.

**Specifications Table**

**Table:** 

Subject area	biology
More specific subject area	promoter sequences
Type of data	text file
How data was acquired	script developed in Python
Data format	Raw
Experimental factors	not applicable
Experimental features	We collected the genome and promoter sequences recognized by *B. subtilis* sigma factors*. The* data (767 promoter sequences) obtained were validated by a software called BacSVM+ which simulates the prediction of promoters in *B. subtilis* bacteria.
Data source location	not applicable
Data accessibility	http://bacpp.bioinfoucs.com/rafael/Sigmas.zip
Related research article	Silva et al. [18].

**Value of the data**

• The data obtained can be used in further studies on gene regulation expression. The regulation of gene expression is essential for bacterial metabolic adaptation to environmental changes, allowing bacterial survival and multiplication.
• Most related papers on bacterial promoters are restricted to Gram-negative bacteria, particularly *E. coli*. The promoters of *B. subtilis described* in this paper allow further research in this area.
• Data on Gram-positive bacteria promoters in the literature are scarce. The process described here can be used by researchers to validate promoters in other bacteria of this type.

## 1 Data

Transcription at a coding region starts when the RNA polymerase (RNAp) enzyme recognizes the promoter region. Promoter regions are conserved DNA sequences that signal and direct the transcription of an adjacent gene or group of genes. Promoters are considered key factors for transcription as they are the initial step in gene expression and part of transcriptional regulation [13]. For this to occur, the sigma factor (a protein factor component of RNA polymerase) must be present on the holoenzyme. The sigma factor determines the specificity of the RNA polymerase on a promoter sequence. After RNA polymerase attachment, the sigma factor is released and gene transcription begins generating an RNA molecule [11].

A typical bacterial promoter is located approximately 70 bp upstream from the starting point of gene transcription. A comparative analysis of several sigma 70 promoters (Gram-negative bacteria) allowed the identification of two consensus sequences: (A) one localized at − 10 bp (5′-TATAAT-3′) from the transcription start point; and (B) another located at − 35 bp (5′-TTGAC-3′). These conserved regions define the affinity of the RNA polymerase complex for a promoter and the accuracy of gene expression. The aim of this paper was to study the promoter regions of *Bacillus subtilis* bacteria and to make a promoter data set available. This bacteria is considered a model organism in laboratory research due to its easy genetic manipulation [10]. The data that were obtained consists of 767 promoters separated into fasta files, each one representing a promoter sequence in *B. subtilis* with a length of 80 nucleotides.

## 2 Experimental design, materials, and methods

**Fig. 1:**
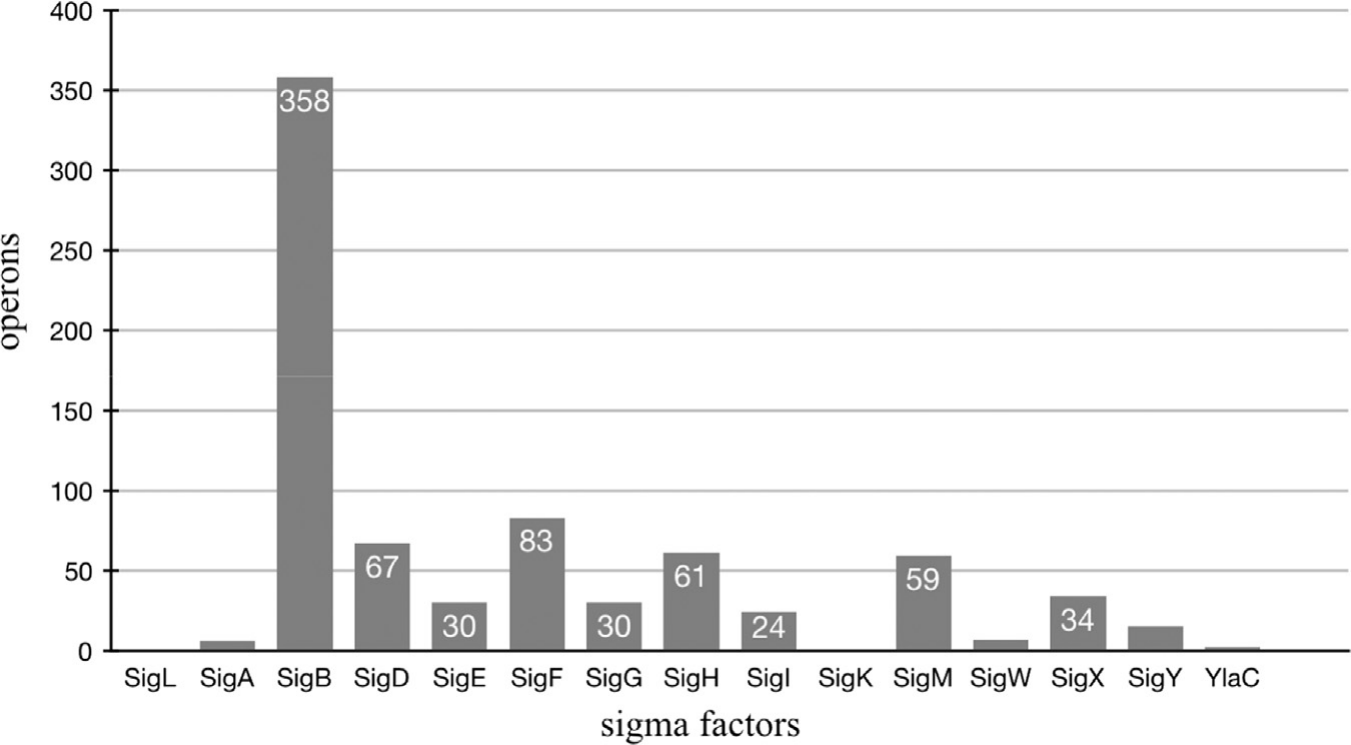
Number of operons per sigma factor of *B. subtilis*. The *X*-axis shows the sigma factors. The *Y*-axis shows the number of operons.

Initially, we collected the fasta file containing the genome of *B. subtilis* from the NCBI (National Center for Biotechnology Information, http://www.ncbi.nlm.nih.gov) database and promoters recognized by their sigma factors from the DBTBS (Database of Transcriptional Regulation in *B. subtilis*) database [17]. This included 15 factors, which we divided into 2 domains: sigma 54 (SigL) and sigma 70 (SigA and others). They are presented in Table 1 with the following informations: ORF (Open Reading Frame), description and *operons*. SigA stands out due to its high number of operons and promoters identified (46.07%). Fig. 1 shows the proportion of each sigma operons.

**Table 1:** Sigma factors of *B. subtilis*[16].

**Domain**	**ORF**	**Description**	**Operons**
sigma54	SigL	RNA polymerase sigma-54 (Sigma L)	6
sigma70	SigA	RNA polymerase major sigma-43 (Sigma A). Essential gene.	358
	SigB	RNA Polymerase sigma-37 (Sigma-B). General stress factor sigma.	67
	SigD	RNA polymerase sigma-28 (Sigma D). Autolytic enzymes; defect in flagellar synthesis.	30
	SigE	RNA polymerase sporulation-specific sigma-29. Processed by SpoIIGA after Tyr-27.	83
	SigF	Synthesized shortly after the onset of sporulation but do not become active until after polar division.	30
	SigG	Control of transcription in the forespore at late stages of sporulation.	61
	SigH	RNA polymerase sigma-30. Non-essential sigma factor involved in expression of vegetative and early stationary-phase genes.	24
	SigI	Temperature-sensitive growth in a null mutant; transcription induced by heat shock in rich medium but not in minimal medium; reduced amount of GsiB protein in a sigI mutant under heat shock conditions.	1
	SigK	Formed by a site-specific recombination event that joins the previously separated spoIVCB and spoIIIC genes into a single cistron.	59
	SigM	Essential for growth and survival in high concentrations of salt; expression maximal during exponential growth and increased in high concentrations of salt; activity negatively regulated by YhdL and YhdK.	7
	SigW	ECF-type sigma factor that mediates the transcriptional response to cell wall stress.	34
	SigX	RNA polymerase SigX.	15
	SigY	RNA polymerase ECF(extracytoplasmic function)-type sigma factor	2
	YlaC	RNA polymerase ECF(extracytoplasmic function)-type sigma factor	1

The data obtained in DBTBS database had the following information: (1) Operon; (2) Regulated Gene; (3) Absolute Position; (4) Location; and (5) Link Sequence. Due to space restrictions, we only present the data obtained for sigma SigL operons in Table 2. This table describes the operon by its gene transcription, transcription start location, genome position (absolute position), binding sequence (red characters are the exact sequence and black characters are the start sequence) and experimental evidence (scientific work that prove the data).

**Table 2:** List of SigL operons [14].

Concerning the experimental evidence for sigma SigL, acoABCL was demonstrated by the mapping of the 5′ extremities of the mRNA by primer extension for the acoA gene and by homology analysis [1]. levDEFG-sacC was demonstrated by both mapping of the 5′ extremities of the mRNA by primer extension for the gene levD [10], the use of a reporter gene, and the disruption of the gene binding factor [7]. Finally, the verification of ptb-bcd-buk-lpdV-bkdAABB, rocABC, rocDEF and rocG came from the mapping of the 5′ extremities of mRNA by primer extension for the gene ptb [8], rocA [5], rocD [9] and rocG [2], respectively.

**Fig. 2:**
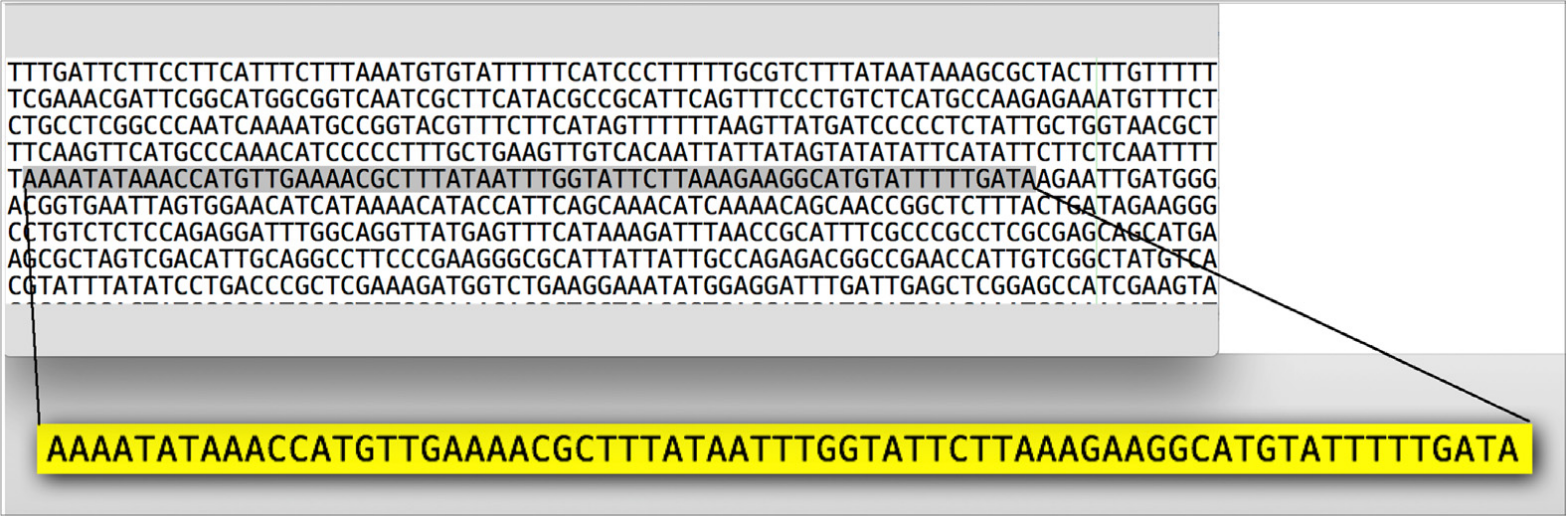
Example of promoter sequence selection from the acuABC operon of SigA in the *B. subtilis* genome.

The FASTA genome file and the promoters obtained were used as input for a program written in Python [15] called searchPromoter.ph (source code in Appendix A). This program was developed to look for promoter regions in complete genomes. The program searched the promoters in the genome FASTA file using the absolute position and if the promoter was not found, the program searched for the sequence. This process was performed on all data obtained. After processing the data using this script, we obtained 767 promoter regions for *B. subtilis*, mostly related to sigma SigA. All data obtained are available for download at http://bacpp.bioinfoucs.com/rafael/Sigmas.zip. Fig. 2 shows an example of how the promoter sequence of the acuABC operon from sigma SigA was selected from *B. subtilis* genome.

To validate the data we found, we developed a software package called BacSVM+ that uses LibSVM library [6] to implement Support Vector Machines [3] for promoter prediction. It receives as input the promoters and returns the best combination of parameters of a LibSVM library to predict promoter regions in the bacteria used in the simulation. Its operation is based on the search for the best combination of LibSVM parameters to maximize prediction accuracy. For this, three steps must be followed during its execution: (A) data preparation; (B) support vectors training; and (C) promoter prediction.

The lack of a user-friendly database could make this first step demanding for users. In this context, the major innovation of BacSVM+ is its data preparation step. If the user does not have the promoters, the program searches (with the python script described earlier) the whole genome for promoters of the respective bacteria. Based on the promoters gathered during the first step, it is possible to define LibSVM parameters and simulate promoter classification.

LibSVM library allows setting a wide range of parameters, as shown in Table 3. Among them, the most important are the cost (*C*) and the gamma (*G*) parameters, where *C* indicates how much the support vectors are penalized when the prediction is wrong. In other words, this is the penalty when points are placed outside the range of correct classification in the hyperplane. On the other hand, the *G* parameter is a way to configure the kernel. In the case of a Gaussian function, this parameter controls the standard deviation function. BacSVM+ allows an extensive search of *C* and *G* parameters by setting a range of possible values.

**Table 3:** Configuration Parameters of BacSVM+.

**Name**	**Description**
gamma (G)	set gamma in kernel function (default is 1/num_features)
cost (C)	only in C-SVC, epsilon-SVR, and nu-SVR (default is 1)
svm type	C_SVC (default), NU_SVC, ONE_CLASS, EPISILON_SVR and NU_SVR
kernel type	set type of kernel function
coef0	set coefficient zero in kernel function (default 0)
degree	set degree in kernel function (default 3)
nu	only in nu-SVC, one-class SVM, and nu-SVR (default 0.5)
cache size	cache memory size in MB (default 100)
epsilon	tolerance of termination criterion (default 0.001)
shrinking	whether to use the shrinking heuristics
probability	whether to train an SVC or SVR model for probability estimates
weight	set the parameter C of class i to weight*C, for C-SVC (default 1)

Finally, in the last step, the user can predict promoter regions and the results can be exported to a text file or a spreadsheet. The architectures performance was evaluated for its accuracy (*A*), specificity (*S*) and sensitivity (*SN*) values, using the following formulas [18].

A=(TP+TN)/(TN+TP+FN+FP)(1)

S=TN/(TN+FP)(2)

SN=TP/(TP+FN)(3)

where: *TP* = promoter sequences classified as promoters (true positives); *TN* = promoter sequences classified as non-promoters (true negatives); *FP* = promoter sequences not classified as promoter (false positives); *FN* = promoter sequences classified as non-promoter (false negatives).

All possible combinations between algorithms (C-SVC, NU-SVC, ONE-CLASS, EPSILON-SVR and NU-SVR) and kernels (LINEAR, POLY, RBF, SIGMOID and PRECOMPUTED) available were made. The cost parameter was set between 0.00390625 and 65,536, with a multiplicative factor of 16. In addition, the gamma parameter was set between 1.52587890625E−5 and 256, with a multiplicative factor of 16. The initial and final values were defined through brute-force tests. The other parameters were chosen according to the default values of the LibSVM library.

The results obtained in simulations with 767 promoters from B. subtilis are consistent with related works found in the literature, thus validating the data gathered. The best combination found was the NU-SVC and C-SVC algorithms with an RBF kernel, leading to a 93.20% and a 95.63% prediction accuracy, respectively. The main innovation of BacSVM+ is in the feature of promoter searching during the data preparation step, allowing the user to use the software even if they do not have promoters and non-promoters examples for running the simulation. Our results can be seen in Table 4.

**Table 4:** SVM results.

**Type**	**Kernel**	**C**	**G**	**A (%)**	**S (%)**	**SN (%)**
C-SVC	SIGMOID	0.0625	1.52587890625E−5	82.04	94.17	69.90
C-SVC	SIGMOID	1.0	0.00390625	85.44	86.41	84.47
C-SVC	SIGMOID	16.0	2.44140625E−4	87.86	88.35	87.38
C-SVC	RBF	1.0	2.44140625E−4	82.04	94.17	69.90
C-SVC	RBF	0.0625	0.00390625	86.41	98.06	74.76
C-SVC	RBF	16.0	2.44140625E−4	91.26	92.23	90.29
C-SVC	LINEAR	0.00390625	1.52587890625E−5	87.86	88.35	87.38
NU-SVC	SIGMOID	16.0	0.0625	57.28	54.37	60.19
NU-SVC	SIGMOID	1.0	0.00390625	93.20	94.17	92.23
NU-SVC	RBF	256.0	2.44140625E−4	95.63	96.12	95.15
ONE-CLASS	SIGMOID	1.0	0.00390625	23.79	0.0	32.67
ONE-CLASS	SIGMOID	1.0	1.52587890625E−5	24.27	0.0	32.47
ONE-CLASS	SIGMOID	0.0625	1.0	48.54	0.0	96.15
ONE-CLASS	RBF	16.0	0.0625	20.87	0.0	26.54
ONE-CLASS	RBF	16.0	1.52587890625E−5	21.84	0.0	30.41
ONE-CLASS	RBF	65,536.0	0.00390625	24.76	0.0	34.46

* Cost (*C*), Gamma (*G*), Accuracy (*A*), Specificity (*S*) and Sensibility (*SN*).

Related works that predict *B. subtilis* promoter regions with Support Vector Machines were found in the literature. Monteiro et al. [12] did not develop their own software. They used the WEKA software that unlike BacsVM+, is implemented in Python and Java languages. In contrast to the 767 promoters used to validate BacsVM+, 112 promoters of *B. subtilis* were used in their research. The accuracy they obtained was lower than the accuracy obtained with BacsVM+, at 76%. Another group developed PePPER as a webserver-based promoter prediction tool (it does not require installation and can be accessed over the Internet), but they did not show results [4]. Finally, TSS SVM [11] analyzes the structural profiles of promoter regions, but it does not focus specifically on the problem of promoter prediction. The authors state that promoter regions are less stable and more rigid than the rest of the genome, but that this is less visible in Gram-positive bacteria such as *B. subtilis*.
