# Supplementary material for: Bacillus subtilis promoter sequences data set for promoter prediction in Gram-positive bacteria
Source: Data Brief. 2018 May 13;19:264–70. doi: 10.1016/j.dib.2018.05.025 (PMC5993011; doi:10.1016/j.dib.2018.05.025)
Supplement: Supplementary file 1 — Transparency document [file mmc1.doc]

**CONFLICT OF INTEREST AND AUTHORSHIP CONFIRMATION**

**PLEASE CHECK THE FOLLOWING AS APPROPRIATE.**

X All authors have participated in (a) conception and design, or analysis and interpretation of the data; (b) drafting the article or revising it critically for important intellectual content; and (c) approval of the final version.

X The Article I have submitted to the journal for review is original, has been written by the stated authors and has not been published elsewhere.

X The Images that I have submitted to the journal for review are original, was taken by the stated authors, and has not been published elsewhere.

X This manuscript has not been submitted to, nor is under review at, another journal or other publishing venue.

X The authors have no affiliation with any organization with a direct or indirect financial interest in the subject matter discussed in the manuscript

☐ The below authors have affiliations with organizations with direct or indirect financial interest in the subject matter discussed in the manuscript:
