# Supplementary material for: Bacillus subtilis promoter sequences data set for promoter prediction in Gram-positive bacteria
Source: Data Brief. 2018 May 13;19:264–70. doi: 10.1016/j.dib.2018.05.025 (PMC5993011; doi:10.1016/j.dib.2018.05.025)
Supplement: Supplementary file 2 — Supplementary material [file mmc2.doc]

**Appendix A. searchPromoter.py source code.**

def openFile(fileName, permission, outputValue):

try:

file = open(fileName, permission)

return file

except IOError as erro:

print ('Error:' + str(erro))

def recordInFile(fileName, text, exitOutputValue):

file = openFile(os.curdir + fileName, 'w', exitOutputValue)

file.write(text)

file.flush()

file.close()

def getLinesFromFile(title, fileName, splitFile):

print(title, fileName)

file = openFile(os.curdir + fileName, 'r', -1)

if (splitFile == 1):

lines = file.read().split('\n')

else:

lines = file.read()

file.close()

return lines

def listFilesFromFolder(folderName, text):

files = os.listdir(os.curdir + folderName)

numberFiles = len(files)

return files

def recordPromoterInFile(sigmaFormated, sigma, folderName, name):

if ('Promotores' not in os.listdir(os.curdir)):

os.mkdir(os.curdir + "/Promoters/")

if (folderName not in os.listdir(os.curdir + "/Promoters/")):

os.mkdir(os.curdir + "/Promoters/" + folderName)

path = os.path.expanduser(os.curdir + "/Promoters/" + folderName)

try:

if (len(sigma) == 80):

filePromoter = open(path + '/' + name + '.promoter.fasta', 'w')

print(sigma + ' - ' + str(len(sigma)))

filePromoter.write(sigma)

filePromoter.flush()

filePromoter.close()

return sigma

except IOError:

print('Error: output file promoter ' + name + '.txt not created.')

def savePromoter(lines, sigma, folderName, index, name, isAbsolute):

posBefore = int((sigma.split('\t')[3]).split(':')[0])

posAfter = int((sigma.split('\t')[3]).split(':')[1])

sigmaFormated = '>' + re.sub("\t", "|", sigma)

return recordPromoter(lines, posBefore, posAfter, sigmaFormated,

folderName, index, name, isAbsolute)

def recordPromoterNone(lines, posBefore, sigmaSize, posAfter,

sigmaFormated, folderName, name, i):

before = lines[(i - posBefore):(i + 1)]

midle = lines[(i + 1):(i + 1 + sigmaSize)]

after = lines[(i + 1 + sigmaSize):(i + 1 + sigmaSize + posAfter)]

if (before.__contains__('\n')):

before = before.split('\n')[0] + before.split('\n')[1]

if (midle.__contains__('\n')):

midle = midle.split('\n')[0] + midle.split('\n')[1]

if (after.__contains__('\n')):

after = after.split('\n')[0] + after.split('\n')[1]

sigma = before + midle + after

if (len(sigma) == 79):

after = lines[(i + 1 + sigmaSize):(i + 2 + sigmaSize + posAfter)]

sigma = before + midle + after

if (sigma.__contains__('\n')):

sigma = sigma.split('\n')[0] + sigma.split('\n')[1]

return recordPromoterInFile(sigmaFormated, sigma, folderName, name)

def savePromoterNone(lines, sigmas, folderName, name, i):

sigmaSize = len(sigmas.split('\t')[4])

position = ((80 - sigmaSize) / 2)

posBefore = int(round(position, 0))

posAfter = int(position)

sigmaFormated = '>' + re.sub("\t", "|", sigmas)

total = posBefore + sigmaSize + posAfter

return recordPromoterNone(lines, posBefore, sigmaSize,

posAfter, sigmaFormated, folderName, name, i)

def recordPromoter(lines, posBefore, posAfter, sigmaFormated,

folderName, i, name, isAbsolute):

if (isAbsolute == 1):

sigmaSize = posAfter - posBefore

else:

sigmaSize = len(sigmaFormated.split("|")[4])

posAfter = 19 - posAfter

posBefore = 61 + posBefore

if (posAfter < 0):

posBefore += posAfter

if (posBefore < 0):

posAfter += posBefore + 1

if (posBefore >= 0):

before = lines[(i - posBefore):(i + 1)]

midle = lines[(i + 1):(i + 1 + sigmaSize)]

after = lines[(i + 1 + sigmaSize):(i + 1 + sigmaSize + posAfter)]

if (midle.__contains__('\n')):

midle = midle.split('\n')[0] + midle.split('\n')[1]

if (before.__contains__('\n')):

before = before.split('\n')[0] + before.split('\n')[1]

if (after.__contains__('\n')):

after = after.split('\n')[0] + after.split('\n')[1]

sigma = before + midle + after

else:

sigma = lines[i:(i + 81)]

if (sigma.__contains__('\n')):

sigma = sigma.split('\n')[0] + sigma.split('\n')[1]

return recordPromoterInFile(sigmaFormated, sigma, folderName, name)

sigmaFiles = listFilesFromFolder('/Sigmas/', 'Number of Sigma Files: ')

genomeFiles = listFilesFromFolder('/Genomes/', 'Number of Genome Files: ')

for genomeFile in genomeFiles:

genomLines = getLinesFromFile('Genome ', "/Genomes/" + genomeFile, 0)

print('_'*100)

number_genome = 1

number_promoters = 1

finalArchive = ''”

for sigmaFile in sigmaFiles:

sigmas = getLinesFromFile('File: ', '/Sigmas/' + sigmaFile, 1)

print('Number of Sigmas: ', len(sigmas))

index = 0

while (index < len(sigmas)):

try:

sigma = sigmas[index].split('\t')[4]

if (genomLines.find(sigma) != -1):

i = genomLines.index(sigma)

try:

sigmaOk = savePromoter(genomLines, sigmas[index], 'GENOME_'

+ str(number_genome), i, str(number_promoters), 0)

if (sigmaOk != ''):

print(str(number_promoters) + ' - ' + sigmas[index] + ' (SIG)')

number_promoters += 1

finalArchive += sigmaOk + '\n'

except ValueError:

positions = sigmas[index].split('\t')[2]

if (positions != 'ND'):

sigmaOk = savePromoterNone(genomLines, sigmas[index], 'GENOME_'

+ str(number_genome), str(number_promoters), i)

if (sigmaOk != ''):

print(str(number_promoters) + ' - ' + sigmas[index] + ' (None)')

number_promoters += 1

finalArchive += sigmaOk + '\n'

else:

positions = sigmas[index].split('\t')[2]

if (positions != 'ND'):

sigmaFormated = '>' + re.sub("\t", "|", sigmas[index])

try:

posAbs1 = int((sigmas[index].split('\t')[2]).split('..')[0])

sigmaOk = savePromoter(genomLines, sigmas[index], 'GENOME_'

+ str(number_genome), posAbs1, str(number_promoters), 1)

if (sigmaOk != ''):

print(str(number_promoters) + ' - ' + sigmaFormated + ' (ABS)')

number_promoters += 1

finalArchive += sigmaOk + '\n'

except ValueError:

sigmaOk = savePromoterNone(genomLines, sigmas[index], 'GENOME_'

+ str(number_genome), str(number_promoters), i)

if (sigmaOk != ''):

print(str(number_promoters) + ' - ' + sigmaFormated + ' (None)')

number_promoters += 1

finalArchive += sigmaOk + '\n'

else:

print('ERROR: ' + sigmas[index])

ads=1

except IndexError:

print(sigmas[index])

index+=1

recordInFile('/Promotors/Promotors' + str(number_genome) + '.txt', finalArchive, 1)

number_genome += 1
